# Mechanisms of collective learning: how can animal groups improve collective performance when repeating a task?

**DOI:** 10.1098/rstb.2022.0060

**Published:** 2023-04-10

**Authors:** Julien Collet, Joe Morford, Patrick Lewin, Anne-Sophie Bonnet-Lebrun, Takao Sasaki, Dora Biro

**Affiliations:** ^1^ Department of Biology, University of Oxford, Oxford OX1 3SZ, UK; ^2^ Department of Zoology, Marine Apex Predator Research Unit, Institute for Coastal and Marine Research, Nelson Mandela University, Port Elizabeth-Gqeberha 6031, South Africa; ^3^ Centre d'Etudes Biologiques de Chizé, UMR 7372 CNRS – La Rochelle Université, 79360 Villiers en Bois, France; ^4^ Odum School of Ecology, University of Georgia, Athens, GA 30602, USA; ^5^ Department of Brain and Cognitive Sciences, University of Rochester, Rochester, NY 14627, USA

**Keywords:** collective learning, individual learning, social learning, collective decision-making, evolutionarily stable strategies, social stability

## Abstract

Learning is ubiquitous in animals: individuals can use their experience to fine-tune behaviour and thus to better adapt to the environment during their lifetime. Observations have accumulated that, at the collective level, groups can also use their experience to improve collective performance. Yet, despite apparent simplicity, the links between individual learning capacities and a collective's performance can be extremely complex. Here we propose a centralized and broadly applicable framework to begin classifying this complexity. Focusing principally on groups with stable composition, we first identify three distinct ways through which groups can improve their collective performance when repeating a task: each member learning to better solve the task on its own, members learning about each other to better respond to one another and members learning to improve their complementarity. We show through selected empirical examples, simulations and theoretical treatments that these three categories identify distinct mechanisms with distinct consequences and predictions. These mechanisms extend well beyond current social learning and collective decision-making theories in explaining collective learning. Finally, our approach, definitions and categories help generate new empirical and theoretical research avenues, including charting the expected distribution of collective learning capacities across taxa and its links to social stability and evolution.

This article is part of a discussion meeting issue ‘Collective behaviour through time’.

## 1. Introduction

Most animals are able to learn, that is, individuals can use their experience to modify their subsequent behaviour, usually adapting to local environmental conditions [1,2]. Much research has also focused on social learning as a fundamental process for cultural transmission and evolution [3,4]. Social learning occurs when an *individual*'s behaviour is influenced by observing or interacting with other individuals, thus learning *from* them, and potentially creating a shared, homogenized pool of knowledge among group members [3]. By contrast, our chief premise here is that we can examine the interplay between learning and sociality from a different angle: that of *collective* rather than individual behaviour [5,6]. We aim to show that although ‘learning’ at the collective level should involve individual and social learning, under certain conditions it can extend much beyond these processes.

For many problems solved in groups, it is possible to quantify ‘collective performance’: the efficiency with which a navigating flock or herd reaches its destination, the success of a group hunt, the defence against predation by a tightly coordinated shoal, or the breeding success of a mated pair, to name a few examples. The collective nature of these measures implies specific mechanisms of ‘collective decision-making’, where several individual actions and preferences have to be integrated into a common ‘collective decision’ or ‘consensus, with potential ‘leaders’ and followers’ if not all individuals contribute equally [7,8]. We use the term ‘performance’ as a reminder that the collective decision is likely to affect individual members' fitness (more on this below). Typically, researchers tend to study collective-level decisions and problem-solving as single events in time [5]. Yet groups can repeatedly face the same collective decisions (e.g. consecutive group hunts), providing members with an opportunity for learning and then using what they learn for collective decisions. Can groups, like individuals, progressively adapt and improve their collective performance by repeating a collective problem-solving task? In other words, can groups ‘collectively learn’ (*sensu* [6])? If so, what mechanisms underlie this capacity, and under which conditions does it emerge?

In this opinion piece, after clarifying below our working definitions, we will first provide some detailed experimental examples of collective learning and their potential mechanisms. These examples come from diverse taxa and behavioural contexts, illustrating many of the complexities arising when trying to compare individual-level to collective-level learning. Second, we will propose a general classification of the different types of mechanisms that could be involved in collective learning, along with empirical examples or candidates. This should highlight that different types of emergent properties (due to interactions between learning members) can give rise to collective learning, involving but not necessarily limited to, the processes of individual and/or social learning. We will end by a synthesis highlighting areas of recent research progress and areas that we believe require further research, while also encouraging particular attention to the potentially strong and deep links between collective learning, social stability and evolution.

**Figure 1.**
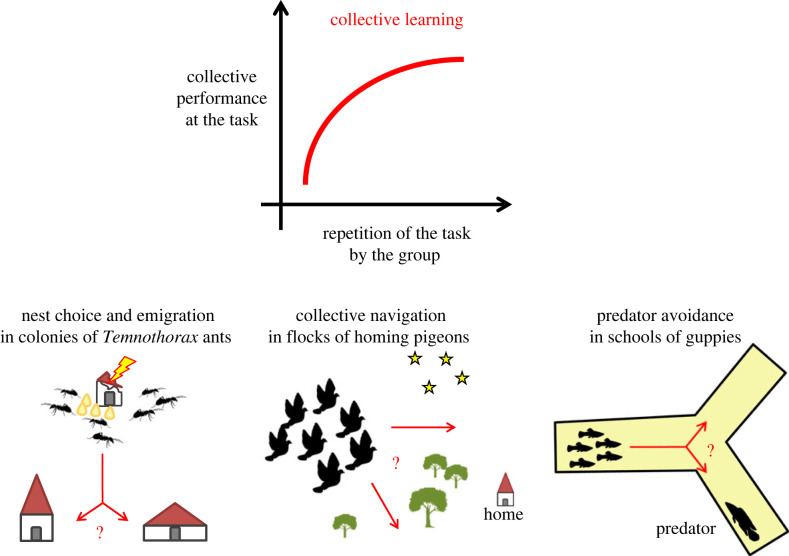
‘Collective learning’, as we define it here, occurs when collective performance at a task (i.e. a measure integrating the behaviour of multiple individuals) changes consistently (usually improving members' fitness) when the group repeats a task (i.e. increases its experience of it). Clear experimental examples include quicker colony emigration to a new nest site after repeated nest destructions in *Temnothorax* ants [9], the development of straighter collective homing routes in pigeons released multiple times from the same site [10] and more accurate collective choice of a predator-free arm in a y-maze in guppies [11]. (Online version in colour.)

Before going further, we will now clarify the terminology we intend to use throughout. We will refer to improvements in collective performance through repeated trials within the life of a group as ‘collective learning’ (figure 1). This definition has three broad, acknowledged implications. First, our definition of collective learning departs from one previously proposed [5] in that it makes no pre-specified assumptions about interactions occurring between individuals. We believe relaxing these assumptions makes the concept much more broadly and objectively applicable by any researcher working at a collective level. We will thus take ‘collective performance’ at its simplest meaning: a measure integrating the contributions of two or more individuals, where these individuals are considered ‘a group’ (regardless of their actual interactions).

Second, in theory, not all learning instances (individual or collective) are necessarily adaptive [12] and could thus arguably *reduce* or *impair* performance. Focusing on positive links to fitness, as implicitly suggested by the word ‘performance’, is not strictly necessary here, but such a stance will both facilitate and emphasize discussions of evolutionary implications. We believe that ‘maladaptive learning’ cases represent the other side of the same coin, i.e. a different type of directed and non-random change of (collective) behaviour arising from experience, but one that should not be favoured by natural selection. We thus hope that the mechanisms discussed here can easily be extended or translated to maladaptive, or even neutral or mixed (i.e. those with different fitness impacts between group members) cases of collective learning. This choice will be further discussed in our final synthesis (§4).

Third, and finally, ‘within the life of a group’ implies that the group persists in time, which might depend on rates of immigrations (and/or births) and emigrations (and/or deaths). To simplify, for the most part, we will thus focus on groups that remain stable in composition across repeated trials; further potential effects of immigrations or emigrations (e.g. [13–15]) will be ignored unless explicitly mentioned and discussed in more detail in the final synthesis.

## 2. Experimental examples of collective learning

### (a) Some case studies and their specific mechanisms

To our knowledge, the first experimental study explicitly focusing on collective learning as we define it examined the responses of ant colonies to repeated nest destructions [9]. If the nest of a colony of ants from the genus *Temnothorax* (formerly *Leptothorax*) is destroyed, individual scouts will start to explore the surroundings to identify potential new nest sites [6]. After finding a candidate new nest site, scouts will transport and/or lead other individuals to its location, until the whole colony has eventually ‘decided’ and settles in the new site. At the collective level, researchers can measure how long it takes for the whole colony to achieve nest migration after initial nest destruction [6] (a ‘collective performance’). How does this time-to-emigration change if the same colony faces repeated nest destructions? Langridge *et al*. [9] experimentally showed that across consecutive nest destructions, colonies became increasingly quicker at choosing a new nest site and at completing the emigration, and they also progressively improved their accuracy in choosing the best site among two candidates (another facet of the colonies' ‘collective performance’). In other words, the colonies showed learning properties (improvement over trials, i.e. with experience) at the collective level: they ‘collectively learnt’. In theory, several distinct mechanisms could explain these observations: recruited scouts could have learned to explore their environment faster; and/or non-scouts may have learnt to follow scouts more efficiently; and/or more individuals may have learnt to readily engage in scouting behaviour after nest destruction, for instance. Later observations concluded that the former hypothesis (more efficient scout exploration) was the main explanation in this case [16].

In some experimental paradigms, it is possible to directly compare the performance of a group with that of a single individual at the same task. By measuring respective changes in collective and lone individual performance across repetitions of the task, we can quantitatively compare collective and individual learning curves and their asymptotes. Groups of domestic pigeons (*Columba livia*) released together from a site away from their home will fly as a flock when returning home, and as such trade off individual navigation preferences against flock cohesion [10]. Across consecutive trials from a single release site, initially unknown to all birds, pigeon flocks refined and improved their collective routes to home, progressively approaching the straight line [10,13]. The collective learning curve (improvement of homing route straightness over trials for birds released in pairs and flying cohesively) appeared strikingly similar to that of individual learning curves obtained from pigeons repeatedly released alone [10,13,17]. It was as if learning of the homing route was no different between lone individuals and members of a flock (see further discussion in [17,18]). Yet from a theoretical point of view this similarity is not trivial. Social interactions that maintain cohesion between flock members could have negatively affected navigational learning, for example by reducing attention to environmental features such as landmarks [19]. Conversely, finding a consensus between individual navigational preferences could have led to flocks quickly averaging out errors around the correct direction (the ‘many-wrongs’ hypothesis; [20]) and thus to a collective learning curve converging more rapidly than individual learning curves [21]. In fact, if these mechanisms were both involved and mutually compensating, similar apparent collective and individual learning curves could emerge despite very different underlying mechanisms.

In some cases, however, collective learning curves can be clearly different from individual learning curves. Shoals of Trinidadian guppies (*Poecilia reticulata*) were experimentally shown to become faster and more efficient across trials (i.e. ‘collective learning’) at choosing the arm of a T-maze that was devoid of predators [11]. In shoals from populations of guppies that had evolved in low-predation environments, the collective learning rate was faster than that of guppies that had evolved in high-predation environments. However, this effect of predator pressure on learning rate was not observed in the learning curves of lone individuals. Hence the level of predation pressure affected collective learning properties but not how single individuals learnt. Further analyses revealed that collective learning in low-predation groups was achieved across trials in part from some individuals becoming more influential on collective movement decisions across trials and in part from an increased sharing of information between members [11]. Here, collective learning properties could not simply be predicted solely from the learning capacities of individuals: the interactions between members and the collective decision-making mechanisms underscored a crucial difference.

A central difficulty is that when observing a collective-learning curve, we have no information on what individual group members each learnt, and whether they all learnt equally. For instance, experiments showed that shoals of zebrafish (*Danio rerio*) could learn to successfully discriminate and collectively choose the ‘correct’ rewarded arm in a T-maze after only a few trials, and indeed did so faster than individuals learning alone [22]. Careful experimental designs showed that most shoals learnt a ‘place response’ relying on surrounding landmarks indicating the correct arm, rather than on a fixed response (e.g. ‘turn left’). However, when individuals from these shoals were later tested alone, there were equal proportions of individual members that had learnt a fixed versus a place response (these proportions matched those of individuals that had learnt alone) [22]. Hence, the learning properties observed at the collective level did not necessarily reflect what individual members had learnt. Furthermore, while in this case what the individual shoal members had learnt within the collective seemed similar to what single individuals learnt, this may not necessarily be the case. Theoretical simulations using groups of machine-learning agents have indeed shown that members of a group can learn very different solutions to a task than identical machine-learning agents solving the same task alone [23,24], as we will see in more detail later (§3c).

### (b) First conclusions: complexities and evolutionary significance of collective learning

As an interim summary, although it may seem unsurprising that collective-learning properties are observed in groups composed of individuals able to learn, the processes underlying collective learning can be complex and also challenging to study. Directly comparing, in the context of the same task, the collective-learning curves of groups with individual learning curves of lone individuals can be a first step; however, such an approach is insufficient in explaining what happens inside the group. Social interactions within the group could strongly affect what and how quickly members learn compared to lone learners; in parallel, what members individually learn might not necessarily be integrated into subsequent collective-decision processes. Moreover, even without learning, pooling the contributions of members may help the group perform better than lone individuals (e.g. the ‘many-wrongs’ hypothesis [20]), so we emphasize that the key observation in our definition of collective learning is an improvement of collective performance *across trials* by the same group. Our examples suggest that for each observed case of improved collective performance across trials, many different mechanistic explanations can be suggested, based on the myriad ways members could interact with one another to reach a collective decision. Classifying these mechanisms could thus help design clearer scientific comparisons between taxa and/or behavioural contexts.

Furthermore, these first few examples also suggest that diverse animal groups from a broad phylogenetic spectrum are indeed capable of collective learning in various types of tasks. This might suggest some evolutionary significance of collective learning. Many animals live in groups, with important evolutionary advantages associated with group cohesion (e.g. see [25]). As a result, the individual fitness of these social animals can be greatly influenced by the outcome of their group's collective decisions [7,26]. It would therefore seem adaptive for the capacity to individually adapt behaviour through learning to scale up to the collective level; in other words, for groups to be able to improve collective performance with experience. One might thus expect collective learning capacities to be widespread, allowing not just individuals but also their groups to adapt their behaviour to changing environmental conditions [2]. Even in solitary animals without direct social interactions, the fitness consequences of individual decisions can partly be dependent on the decisions of others [27,28]. As a result, at least in some cases, it can make sense to test if these ‘groups’ of solitary neighbours improve their collective average performance across consecutive measurements over their lifetime (e.g. to better assess conservation chances in a population [27]). In the next section, we will thus detail and classify potential mechanisms for collective learning, to hopefully facilitate their systematic investigation in diverse animal groups.

## 3. Mechanisms of collective learning: a classification

**Figure 2.**
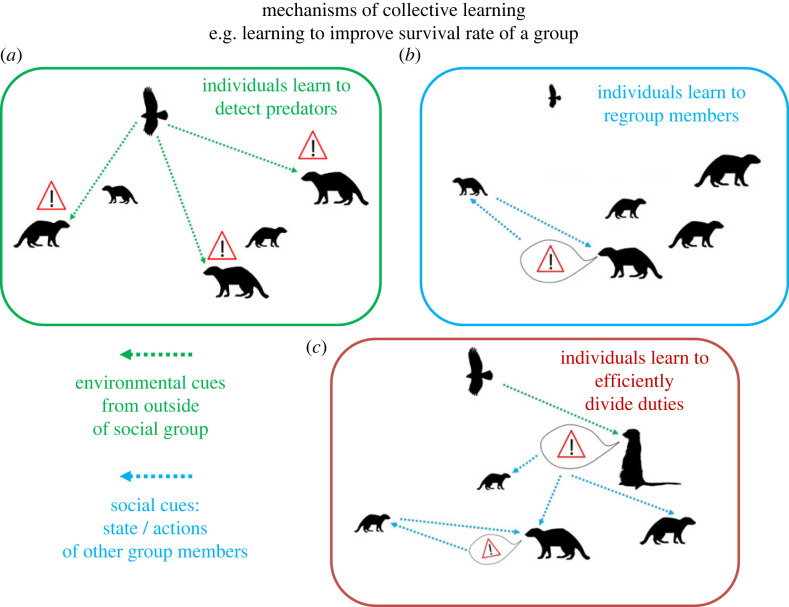
Schematic representation of three distinct but non-mutually exclusive mechanisms through which groups could increase collective performance through experience, illustrated with the repeated collective task of not losing a member to a predator encounter. (*a*) Individuals may each progressively learn to recognize/detect predators and thus quickly get to safety; (*b*) individuals may learn to detect and reduce risky behaviours from other members to ensure they all get to safety, and/or they may learn to attend to and correctly interpret warning calls; (*c*) individuals may progressively specialize into subtasks (e.g. predator versus social vigilance) and thus avoid interference and/or increase complementarity between individuals with different specializations. (Online version in colour.)

As we have seen, there are several potential mechanisms through which a given group can improve its collective performance through experience. At first sight, these mechanisms could appear to strongly depend on the species, social structure and/or collective task considered. Yet we have identified three distinct categories of mechanisms that could enable stable groups to improve performance when repeating a collective task (figure 2). First, individual members could learn to individually improve at solving the task on their own. Second, individual members could learn about other members and improve how they respond to each other while performing the task. Third, individuals could learn to adjust their contribution to the task so that it better complements the contributions of other members. As an analogy we can think about a band of musicians learning to improve their performance at playing a particular song: members could each improve their own playing of their part; members could improve how well they listen to and respond to each other and play in better synchrony; or else members may adjust which instruments are played in the band for an improved overall effect.

As we will see, these three categories of mechanisms overlap (that is, some observed empirical processes could fit several categories simultaneously), but the essential point is that they overlap only partly: some cases of collective learning can only be assigned to one or two categories of mechanisms and not to the other(s). By mainly focusing investigations on one category of mechanism (as we will argue is the case of the current scientific literature explicitly addressing collective learning), we could fail to identify many cases of collective learning. Moreover, each category has specific implications and predictions as we will progressively detail.

All three categories assume individual learning capacities by group members (which for simplicity we will here equate to associative learning processes [1]) and might (or not) involve social learning processes (i.e. learning *from* others, which can also be explained in associative learning terms [29,30]). However, we will show that collective learning can potentially involve further emergent properties due to interactions between learning agents, that are not necessarily predictable by considering individual or social learning properties alone (see also [31,32] on such claims). We also note here that some simulations showed that collective behaviour can be influenced by the history of the group (i.e. a collective memory effect, revealed by ‘hysteresis’ analyses) even when members had no learning capacities implemented [33]. However, to our knowledge, this phenomenon has received relatively little interest from biologists since its initial report, and its biological significance is thus very poorly known. It is unclear if it could have long-lasting effects on collective behaviour and/or individual fitness, especially in groups of real animals who generally do have individual learning abilities [1]. For all these reasons, we will not further explore this type of process here, but it might be worth for future studies to consider its potential impacts in more detail.

### (a) Members learn to better solve the task on their own

Perhaps the simplest way for a group to improve its collective performance is by each member learning to individually solve the task better and thus improving its contribution to the group's pool of contributions. If all members' input improves, the collective performance should indeed improve, regardless of whether the collective decision emerges from some form of averaging (weighted or not) or from extracting a single solo contribution from among group members. We argue that this occurs during collective learning of homing routes in pigeons: virtually all individuals within the group seem to learn the route home through collective trials. Indeed, when they are separated from their group they still perform well on their own, at levels similar (albeit not strictly identical) to their group's previous performance [18,21,34]. Similarly, in the zebrafish experiments cited above [22], it seems that most members of the shoal learnt to enter the correct rewarded arm, so even though not all members acquired the same mechanism for selecting this arm, at the collective level the group learnt the correct choice. As these two case studies reveal, testing group members alone, after the collective-learning phase, can be instrumental in revealing what they each learnt, although it may not always be necessary.

Some cases of collective performance emerge from different individual members performing very dissimilar, specialized contributions (sometimes referred to as ‘division-of-labour’ [35,36], or ‘social roles’ or ‘niches’ [37–39]). The example of nest destructions and emigrations in *Temnothorax* ants mentioned above [9] falls into that category: only a subset of the colony's individuals act as ‘scouts’ that explore the environment for options, and at any given time, some recruited ants are passively carried while others more actively follow ‘demonstrators’. In such cases, no single individual performs the entire task on its own. However, even then it is still possible to test whether individual members improve their individual performance, across collective trials, at their subtask. This happened in the case of ants: much of the collective-level increase in performance across trials was due to individual-level improvements in exploratory performance [16]. We shall return later (§3c) to how such roles might spontaneously emerge within a group and how this could contribute to increases in collective performance across trials, but no such processes seemed to be involved in the case reported in Landridge *et al.* [9,16].

At its most basic expression, this first category of mechanism might thus appear to involve little in the way of emergent collective-level properties. Under previous definitions [5], some authors might actually disregard it as a case of ‘collective learning’. Yet if we consider inter-individual heterogeneity in behaviour [40], in experience [13] and/or in learning capacities within the group [41], emergent properties may be likely to occur, even if they are difficult to study. First, members' improvement at individually solving the task may be facilitated by social interactions (social learning), for instance by copying successful actions (imitation or emulation) or more simply by increasing the individual's exposure to relevant cues (local or stimulus enhancement) [3,30]. Such social learning might be additionally facilitated if the group contains members with previous experience of the task (e.g. [13,34,42]), but this would imply a change in group composition, which we will discuss in more detail later (§4b). However, social learning processes might also occur in a group of initially all-naïve individuals, if some members learn faster or fortuitously make better contributions that are recognized as more efficient and then (socially) learnt by other members (a form of ‘information pooling’ [26,42,43]). Indeed, the mechanism could even encompass simple social facilitation: the mere presence of conspecifics may lower individuals' neophobia and time devoted to vigilance [25], and in turn increase their attention to the relevant elements of the task. In homing pigeon flocks for instance, some of these social learning processes seem involved in collective learning of navigational routes [13,43].

A second simple emergent property may arise if the contribution of individual members to collective decision-making is modulated by those members’ knowledge of the task at hand. For instance, if the more knowledgeable members tend to act as leaders, the group's performance could increase faster than the average performance of its members. In homing pigeons again, links between knowledge and leadership have sometimes been observed, although not under all conditions [43–46]. More generally, there is a rich literature on the links between leadership and individual knowledge in different animal groups (e.g. [47–49]), the review of which extends beyond the present manuscript.

These emergent properties might often be challenging to demonstrate in stable groups, especially if they occur on very small spatio-temporal scales within collective trials [50]: identifying who learns from whom, to what extent information is pooled among all members, or how knowledge influences leadership in real time across trials will not always be possible when individuals are constantly interacting with each other. However, several non-mutually exclusive approaches can help reveal them. First, insights can be gained by careful monitoring (e.g. [11,51]) and/or experimental manipulations of group composition (especially heterogeneity in members' knowledge; e.g. [13,45,49]) and the resulting effect on leadership and collective performance within or across trials. Second, the use of simulation models can also enable the comparison of predictions with or without some of these processes (social learning, leader–follower roles, etc.; e.g. [51]).

Despite these empirical difficulties, currently much of the research explicitly addressing collective learning seems to focus on these mechanisms [5], as two now classical research avenues are merging (animal cultural behaviour on the one hand, and mechanisms of collective decision-making on the other). It also appears that many of the most explicit cases of collective learning reported, and presented in the previous section, rely in large part on this general mechanism of individual members improving at the task. Nonetheless, other categories of mechanisms are plausible, and we turn to them below.

### (b) Members learn about each other

When considering individuals within a group, we have to examine the possibility that individual behaviours can be a direct response to perceptions of neighbours (i.e. their state and/or actions), what we could call ‘direct interactions’. For instance, simulation models of collective movements can recreate complex dynamics of flocks or shoals only when members account for neighbours' positions around them to decide on their own direction and speed [33]. While it is generally assumed in many collective movement models that the rules governing these direct interactions are fixed (e.g. [23,24,52]), in theory they could be influenced by the learning capacities of members. In associative learning terms, individuals could form an association between what they perceive about others and how best to respond (i.e. not simply learning *from*—i.e. social learning—but also learning *about* each other). In turn, learning to interact (directly) with other members might contribute to improve some measure of collective-level performance.

This mechanism could of course extend beyond the case of collective movements. In vampire bats (*Desmodus rotundus*), groups can improve the average survival rate of their members (our ‘collective performance’) through a form of non-kin cooperation (sharing blood meals), which works as a collective mutualistic insurance against stochastic individual foraging success [53]. Recent studies suggest this altruistic behaviour develops progressively between any given pair, by establishing a form of trust relationship specific to the partner: individuals are more likely to offer a blood meal to individuals they have previously roosted with and that groomed each other; it is also less likely to happen again towards non-reciprocal individuals [53–55]. If we imagine a newly formed group of vampire bats, all initially naïve to each other and to foraging, we could in theory expect the average survival rate of the group to increase through time and across foraging attempts, as members learn to trust each other through their interactions. Importantly, such collective learning could arise even if individuals do not get individually better at foraging. Here, collective learning could arise from individuals learning about each other rather than about solving the task on their own.

Collective behaviours that involve some level of coordination between members are likely to provide other candidates for this mechanism of collective learning (e.g. [56–58]). In particular, song duetting in passerine species could become a powerful example to study such individual and collective learning mechanisms in more detail [58]. In song duetting birds, both males and females vocalize to produce a coordinated song (either in synchrony or in highly precise alternance, sometimes with sex-specific phrases). Evidence suggests that like more classical singing in passerines, song duetting species also have to learn their repertoire, and presumably also the repertoire of duet-partners and how to coordinate their responses. However, many of the specific mechanistic questions raised by the coordinated nature of song duets remain to be investigated [58].

Another way of identifying candidates would be to start from some of the many known and diverse examples of individuals forming mental representations about others. Conspecific individual recognition and use of that skill during interactions has been shown in various organisms [59–61]. Some predators, including raptors or dragonflies, can anticipate the behaviour of their prey (such as their escape trajectory [62,63]), and it might not be unrealistic to imagine similar anticipatory capacities targeted at conspecifics in dense, fast-moving flocks or shoals. In monogamous pairs of birds, there is evidence that individuals can account for the physiological state of their partner to adjust their parental effort [64], and in primates, there is a long tradition of research on theory-of-mind (being able to represent others' state of perception or knowledge; [65]). Exploring, on the one hand, the influence of learning in the context of these capacities, and on the other hand their consequences on collective performance across trials, would then provide confirmation of the role of learning about other group members in collective learning.

In all the candidate examples we searched, we never found all these elements empirically demonstrated together. For instance, in monogamous birds, it is a common observation that pairs improve their breeding success across joint breeding attempts (e.g. [66–68]; a form of collective learning by our definition). In theory, this improvement could arise if individuals progressively *learn* to better account for their partner's state to adjust their parental behaviour [64]. However, to our knowledge the mechanisms for the progressive improvements of pairs' breeding success across attempts are still largely unknown (e.g. see [69,70]).

When collective learning is shown in a system, evidence that it emerges from members learning about each other could be obtained indirectly, by manipulating group composition during the collective learning phase. For instance, if individuals were to always behave as part of a group during the collective learning phase, but with ever-changing partners, would they still show the same collective learning properties (e.g. see [70])? Even in non-manipulated stable groups, with modern tracking technologies enabling us to record fine behavioural responses of numerous individuals at a time, it could also be possible to statistically assess whether individuals become, for example, faster to respond to their neighbours across trials [11,71]. This is indeed what is suggested by detailed analyses of the predator avoidance experiments in guppies [11]. Finally, modelling simulations that switch this process of learning about others on and off could also enable us to develop further testable predictions. Yet to our knowledge this latter avenue has seldom been explored by modellers: even when learning rules are implemented in biologically inspired models (e.g. [23,24,52]), all models we know of implement direct interactions as a fixed rather than as a learnable and tuneable rule: learning is directed towards other components of the behaviour. It should be noted that this is not the case in Artificial Intelligence (AI) studies [72], which may thus offer up useful insights for biologists.

To sum up, this mechanism of learning about others is at least partly distinct from the mechanism of members improving their individual performance at the task (§3a). Indeed, we can theoretically imagine a scenario where the improvement of group performance mainly relies on members learning to better respond to each other, such that if members were to be extracted from the group and tested alone, they would perform poorly (a form of inter-dependence). This would be opposite to our prediction in the previous section where testing members alone would reveal they improved at solving the task alone. We acknowledge that the distinction between these first two categories may become blurred when the collective performance explicitly integrates a social dimension (e.g. synchrony), and as such cannot be solved alone (in contrast with navigation, hunting, etc.). Moreover, there is a third category of mechanism, which also predicts potential inter-dependencies between members (and thus poor individual performance of members tested alone). The distinction between the second and the third category of mechanisms will be developed in the next sub-section.

### (c) Members learn to better complement others

Collective performance is a mathematical function integrating the behaviours of several individuals into a single measure [8]. Depending on the nature of this function, collective performance might be higher than any of the individual members’ contributions on their own, a phenomenon that exemplifies ‘collective intelligence’ [13,20]. A well-known mechanism for this is provided by the ‘many-wrongs’ principle: a group averaging its members' individual estimates (for instance of a correct navigational direction) will statistically increase its accuracy (if individual estimates are independent from each other and unbiased), as the random errors in individual estimates are averaged out [20]. The many-wrongs process can for instance be observed in some homing pigeon flocks [21]. By contrast, members with high individual efficiency when they are on their own may form poorly performing groups when put together [73,74]. In other words, members may complement each other (the collective performance is then better than the individuals’ contributions), or they may, on the contrary, ‘interfere’ with each other (the collective performance is then worse than members' individual performances). The corollary is that across repetition of a collective task, if individual members somehow learn to better compensate each other or if they learn to reduce interferences among them, the overall collective performance will improve across trials. We refer to this effect as collective learning through improved complementarity. We will see that it can emerge spontaneously from simple individual (and/or social) learning rules. We will further show that it can arise even if members do not get individually better at the task or if members do not explicitly learn *about* others.

A pioneering study by Kao *et al*. [23] first exemplified this case through a modelling simulation placing individuals in a particular trade-off, where collective accuracy could reach higher levels than that possible for individual accuracy (based on the many-wrongs principle). It showed that neural networks placed in a group progressively learnt to rely on the many-wrongs mechanism to gain higher collective-estimate accuracy, rather than to improve their individual estimates at the expense of the collective accuracy. When learning reached an asymptote, these groups were thus composed of individual members that would perform more poorly than identical but lone learners [23], although as a group they performed better.

A later example, perhaps more intuitive, is provided by a different modelling simulation [24]. There, neural network agents were paired to collectively navigate. Each neural network independently proposed a direction, in the first trial chosen randomly. In the experimental treatment of interest to us here, the two ‘individual’ directions were then averaged to determine the pair's collective direction (cohesion was forced). The magnitude of the difference between this collective direction and the ‘correct’ direction (arbitrarily chosen but constant across trials) was used to modulate the reinforcement of each neural network of the pair in its individually proposed direction. Across trials, the collective direction of most of these pairs converged towards the correct direction (collective learning) [24]. However, in most of these pairs, none of the individual neural network members converged to individually propose the correct direction. Rather, each pair stabilized at an idiosyncratic equilibrium where a constant clockwise error of one neural network was almost perfectly compensated by a constant, opposite anticlockwise error of its partner [24]. Hence across trials, even if the individual members did learn, none learnt to better solve the task on their own: they would have poorly performed alone. Note that this might affect our methods to efficiently detect individual learning, when individuals act within a collective.

Other simulation studies have reported similar improvements of collective performance through individuals spontaneously increasing their complementarity across trials (e.g. [27,75]). Importantly, in these simulations, members could not track or directly account for the individual state or behaviours of their partners. Individuals could only rely on previous experienced outcomes (as reinforcement for learning), but in contrast with lone learning, these previous outcomes could potentially be influenced by other, sometimes numerous individuals. Interactions with these other individuals were therefore ‘indirect’. This category of mechanisms is thus distinct from the previous one in that members do not necessarily learn about each other, i.e. they do not learn to associate their choice of behaviour to perform with a cue perceived about another member. The distinction may become particularly important when considering very large groups (in terms of numbers of individuals, and/or in terms of spatial extent [26]). To perhaps illustrate this even more clearly, we can consider a simulation where individuals might never encounter each other [27].

Under some environmental conditions, simulated individuals only using individual learning and spatial memory to forage for patchy, depletable resources can progressively and spontaneously segregate over the landscape in distinct home ranges [76]. This occurs with no rules for social interactions (nothing happens when two individuals meet, by chance), and no rules for territorial behaviour (no marking or defence of borders). Spatial segregation occurs simply because individuals are not reinforced when encountering a patch already depleted by another individual [76]. In turn, at the collective level this allows the population as a whole to exploit a progressively larger share of the landscape's resources and to reach higher abundance (i.e. an increase in carrying capacity), corresponding to a case of collective learning [27]. In this case, individuals also benefit from the situation, as they escape competition and improve their individual consumption rate (i.e. this case could fit both categories 1 and 3 when considering individual- and collective-level foraging success) [27]. However, the limits of their home ranges are arbitrary and there are no intrinsic differences between the different home ranges. In other words, individuals did not learn to exploit more productive parts of the landscape; instead, they learnt to better divide it among them, without direct interactions.

So far, we have restricted examples to modelling simulations. The sets of models explaining spatial segregation between foraging individuals have received strong empirical support in bats [77] and in seabirds [78]. In seabirds, this effect was observed after adding further complex processes of social information transfer within subgroups: segregation at sea in seabirds was observed mainly between individuals from different rather than from the same breeding colony [78]. This exemplifies that collective learning through complementarity can also involve social learning. A distinct simulation model sharing similar spatial properties was also developed to explain empirical observations in humans playing a cooperative game online without direct communication or tracking of the partner's actions [75]. It is likely that many other empirical examples exist of these processes, but their indirect nature makes them very difficult to disentangle without the help of simulation models.

A common, recurrent pattern observed with this third category of mechanism is that in almost all cases individual members progressively specialize their behaviour: neural networks specialize on a specific direction, either clockwise or anticlockwise [24], and individuals segregate over a landscape [76–78]. One potential way to empirically test for collective learning through improved complementarity between members could thus be to examine how members' specialization changes through time, and how this affects overall collective performance. To assess changes in specialization through time, one can look at the change across trials in within- and in between-individual variability in behaviour among group members [35]. There are many empirical and theoretical studies that have investigated how social roles (e.g. leader versus follower [79]), division-of-labour [36,80,81] and/or personalities [39,82,83] can spontaneously and progressively emerge within collectives, but the link to a progressive increase in collective performance through this mechanism seems to have been rarely addressed empirically [84]. In eusocial insects, for instance, while division-of-labour is ubiquitous and the current literature favours mechanisms of self-emergence within colonies, an empirical link from the progressive emergence of division-of-labour to any colony-level performance is still lacking [81,85].

This ‘specialization test’ among members might also help highlight a further distinction between ‘learning about others' (§3b) and ‘learning to complement others’ (this sub-section). In the case of vampire bats discussed in §3b, it can be argued that members learn to complement each other. Indeed, when one member has poor foraging success, the others compensate for it; hence, the gain at the collective level is through the consensus function and not necessarily through individual improvement. As we saw in the previous section, it also occurs through learning about each other (i.e. the case fits both categories 2 and 3). Yet in the vampire bat case we would not necessarily predict a progressive specialization of individuals into different roles. In fact, across trials, individuals should have roughly symmetrical contributions in terms of feeding others or being fed by others: reciprocity seems a requisite to establish and maintain trust relationships [53]. As another key distinguishing prediction, if for any reason one member suddenly changes its behaviour (or disappears) during a trial T compared to previous trials, if other members had learnt about this specific individual (§3b), they should immediately adjust their own behaviour in the same trial T (since the cue to which their behaviour responds has changed). However, if members had learnt to complement each other without learning about the others (this sub-section's mechanism), it should take longer (i.e. at least another trial) for them to change their individual behaviour. Hence, we have our three, partly overlapping yet distinct categories of mechanisms.

## 4. Synthesis and key areas for future research

Our survey of empirical examples, including clear experimental results from taxa as diverse as ants, pigeons and fish, suggest that collective learning capacities could be widespread across animal groups. To facilitate their systematic investigation, we have proposed classifying the mechanisms of collective learning into three broad categories: members learning to better solve the task on their own; members learning about each other to improve how they directly interact; and members learning to act so as to better complement others' actions. We highlighted how these mechanisms could be tested and distinguished. Below we will suggest a broad roadmap for future research questions, arising from our definition of collective learning and our proposed categories of mechanisms.

First, we emphasize once more that our discussion of collective learning mechanisms suggests it may be misguided and counterproductive to try to fit any given empirical or modelling case of collective learning in a single of the three categories of mechanisms described. The three categories overlap partly, and/or can jointly operate. Rather, these categories can help to consider broad sets of alternative mechanistic hypotheses when collective learning is observed. Indeed, each category of mechanisms presents distinct consequences and predictions to be tested. We provided various candidate examples where such investigations might prove fruitful. The definitions we adopted also highlight two further and broad avenues of research that we now turn to: when should we expect collective performance to have (or not have) the capacity to improve over time, and thus how might collective learning capacities be distributed across animal groups; and what influence can emigrations and immigrations (i.e. ‘social instability’) have on the processes we described, and on collective learning more generally?

### (a) The distribution of collective learning capacities, or why collective learning may often fail to emerge in groups

We focused our discussion of mechanisms on cases where ‘collective learning’ occurred, i.e. was detected through an improvement of a group's performance across trials. Yet we acknowledged in the introduction that not all learning is necessarily adaptive. In fact, because our definition of ‘collective performance’ is very broad (any measure integrating the behaviour of several individuals), trivially we expect that only some collective measures should increase across trials. This leaves open the question of the actual distribution of collective learning capacities across species, groups and/or tasks. Studies reporting collective learning failing to emerge in biological groups, and explaining why, would thus be essential to better map these capacities.

To explore when and how collective learning fails, biologists might find inspiration from empirical works of AI engineers. For decades now, AI researchers have been striving to develop teams of robots each capable of individual learning, and trying to solve cooperative tasks together (e.g. see an early introductory review of hundreds of such studies in [73]). Many of the challenges the robots and their engineers faced, reviewed in Panait and Luke [73], will probably also be relevant for groups of learning animals. Indeed, in many cases, teams of robots did not improve at solving the cooperative task [73]. Robots, however, can only be imperfect models for animal groups, primarily because they do not have a long evolutionary history, and are often designed to complete a specific task, rather than to balance and trade off various needs related to survival and/or reproduction [12,86,87]. Therefore, there is still ample scope for biologists to assess the factors influencing when animal groups should fail or succeed in collective learning. We provide below some more detailed outstanding questions and hypotheses.

**Figure 3.**
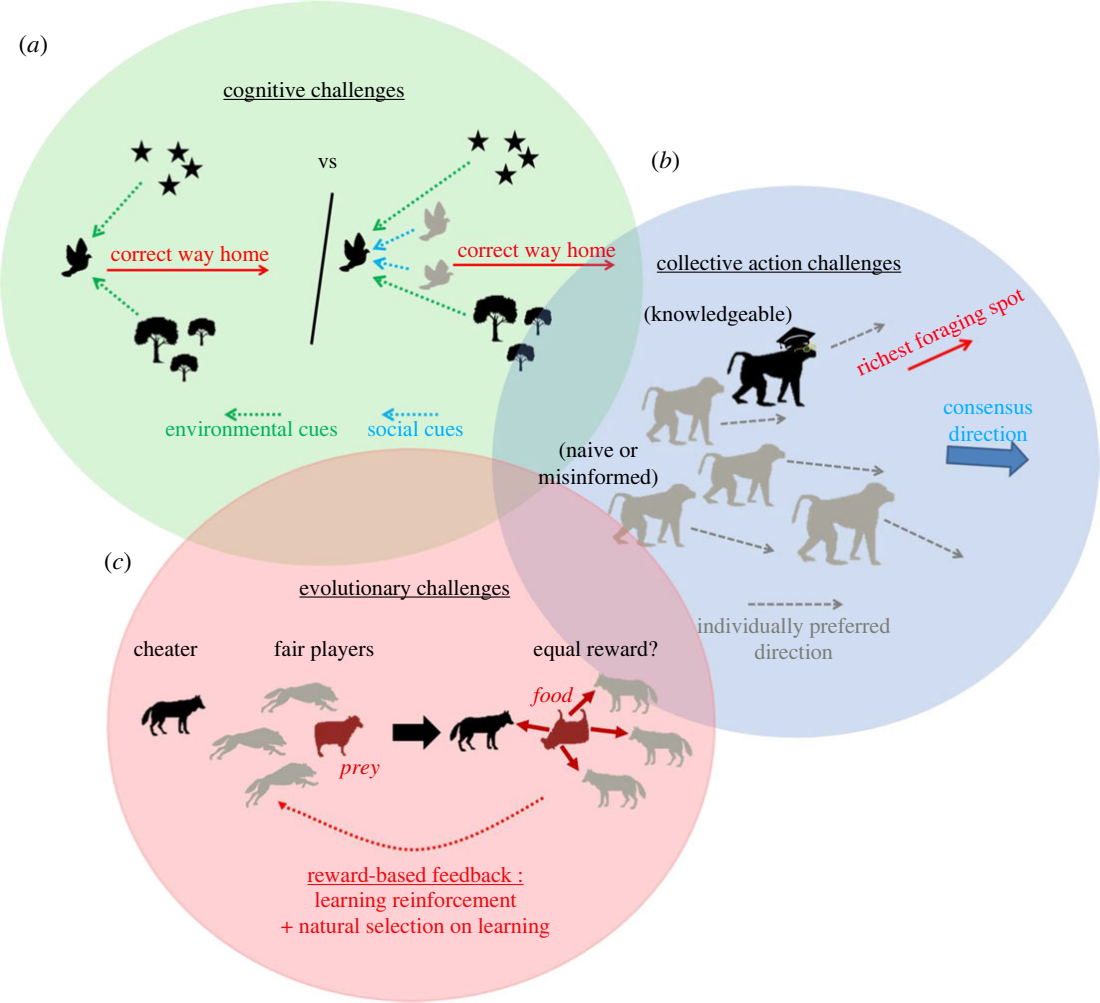
Schematic representations of non-mutually exclusive constraints on collective learning capacities which might help predict its distribution in animal groups: (*a*) the individual cognitive processing of information may be more challenging when several individuals, rather than only a single individual, contribute to a task; (*b*) what individual members learn and/or know does not necessarily influence the collective decision; (*c*) conditions for the emergence of collective learning need to be evolutionarily stable strategies, successfully traded off against other biological functions, to persist across generations. (Online version in colour.)

We suggest there are three broad types of challenges for the emergence of collective learning in animal groups composed of individuals capable of learning (figure 3), which might then help to predict the distribution of collective-learning capacities in groups. The first type of challenge is *cognitive*: being part of a collective might alter what or how fast individual members learn. If for any reason individuals fail to learn efficiently when they are within a group, the collective should not learn either. Biological simulation studies indeed often reported slower learning of collectives and/or of members of collectives, compared to lone individuals [23,24,76]. In some cases, individual members (and thus their groups) even failed to converge on a solution [24]. Broadly summarized, many such effects can be due to social cues and social interactions adding noise around relevant signals and slowing down the learning of relevant cues or actions by individuals. Some theoretical and empirical hypotheses for these processes can be found in the previously cited review on cooperative robotics (non-stationarity of the environment, i.e. ‘co-learning’; wide joint-solutions space to be explored by trial-and-error; importance of the reward system for learning reinforcement, etc.) [73]. Other, more biologically oriented hypotheses can be found in the psychology and neuroscience literatures [1], reporting various types of competition between cues during perception and processing (e.g. limited attention [19,88], over-shadowing or blocking [1]) which interfere with or slow down learning.

Yet several empirical studies in animal groups found an opposite, positive effect of the collective context on learning rates, or no effects at all (e.g. [10,89,90]). More work is thus needed to elucidate which of the hypothetized cognitive challenges suggested by theory and simplified simulations apply to real animal groups and/or which solutions may have evolved for animal groups to overcome these. In turn, this might help better predict the distribution of collective-learning capacities across taxa. It might be that collective learning is more likely to occur in animals whose brains compartmentalize the processing of social from physical cues (e.g. through brain lateralization [91–94]), or in animals with generally enlarged neural processing capacities (e.g. see ‘the social brain hypothesis’ [95,96]).

The second type of challenge is *collective*: even if individual members learn, what they learn may not be integrated into subsequent collective decisions. We previously mentioned (§3a) existing research on the links between knowledge and leadership, for instance (e.g. [13,45,48]). Other approaches have been linking social network properties (size, modularity, etc.) to rates of information transfer (social learning) between individuals and to the efficiency of information pooling at the collective level (e.g. [97,98]). It is thus likely that not all social structures will favour the efficient integration of members' newly acquired knowledge into collective decisions. Within non-human animal groups, empirical work suggests that collective decision-making processes and/or social networks can vary extensively with species, populations and/or environmental or social contexts within populations (e.g. see the case of travelling baboon troops [99–101]). It is thus possible that the distribution of collective learning capacities in animal groups would best be predicted by social organization rather than cognitive capacities. The question of efficiently integrating members' knowledge at the collective level also has a long history of research in various human sciences (e.g. [102–106]), but it is largely beyond our expertise to assess to what extent the methods and/or conclusions therein could transfer to non-human animals.

The third type of challenge is linked to evolutionary constraints, potentially interacting with cognitive and collective processes. Collective learning might emerge by chance in a group where members can learn but impose important fitness costs on some members on which it is reliant, such that it might not persist across generations, due to natural selection. The interactions between genetic and learning processes in giving rise to evolutionarily stable strategies are still poorly known [12,87]. Moreover, the evolution of learning capacities themselves has mainly been studied from an individual rather than a collective perspective so far [87,107]. In their review of cooperative AI research [73], Panait and Luke suggested that one key to successful cooperation between learning robots was to implement the correct reward scheme to better align individual and collective objectives. It would be particularly valuable to explore whether similar considerations may be relevant to predicting the distribution of collective-learning capacities in animals, through an analysis of the fitness costs and benefits to individual members.

### (b) Links between collective learning and group instability

So far, we mostly focussed our discussion on stable groups, where improved performance across trials is not linked to altered group compositions. This arbitrary choice was made to focus on the already complex links between learning at the individual level and the processes of collective decision-making. Yet in many animals, groups remaining stable across trials may be the exception rather than the rule [108]. Moreover, theory and empirical observations both suggest that emigrations and immigrations (and/or birth and death) can play pivotal roles in shaping the collective performance of groups across time. Overall, changes in group composition can indeed alter the ‘sum’ of knowledge present within the group [99,109–111]; they could also at least temporarily disrupt collective organization and the efficient integration of relevant knowledge among members, but they might on the other hand favour innovations, as strongly suggested empirically [13–15].

A first clear observation is that most of the distinctions between the three different categories of mechanisms we proposed will be best revealed by sudden changes in group composition (see §3). In particular, collective learning based on ‘learning about others’, or based on ‘learning to complement others’, could be highly sensitive to group disruptions. These two categories of mechanisms create inter-dependencies between group members, so that their collective performance relies on members remaining together, although in large groups, redundancy of social roles among individuals might partly alleviate this effect. Our short summary of potential challenges to collective learning (§4a) also suggested that in many cases being part of a collective might slow down the learning rate of groups compared to lone individuals. Long-term group stability could thus be of paramount importance to learn about others and/or to learn to complement others. In fact, even in homing pigeons where collective learning of navigational routes seemed not to involve such inter-dependencies, social disruptions caused large drops in collective performance which took several trials to recover to (and eventually overtake) pre-disruption levels [13].

Based on these considerations about sensitivity to social disruptions, one could hypothesize that ‘learning about others’ and ‘learning to complement others’ should primarily be observed in phylogenetic lineages with a long history of high group stability. Yet things might not be so simple: the modelling simulation studies we reviewed suggest that at least ‘learning to complement others’ can spontaneously emerge from very basic individual learning abilities [23,24,76]. Since most animals can individually learn, many collectives may spontaneously come to rely on inter-complementarity between members. More work is thus needed to evaluate how often and to what extent repetitive collective performance in animal groups is sensitive to group disruptions. More theoretical work is also needed to understand how this might have affected the coevolution of learning and social traits in animals [25,87,107].

Positive effects of recruitment on collective performance could also be actively leveraged by group members. At least in humans, groups and institutions can rely on selective recruitment of members into the group (as well as selective exclusion), based on their expected capacity to improve collective performance (through one or several of the three mechanisms of collective learning we proposed). It is unclear if such selective recruitment processes could also apply to non-human animal groups, although at the level of pairs or very small groups, this is reminiscent of assortative mating and/or non-random coalition bonds within groups [112,113].

At a time when the planet is undergoing major global changes, the sensitivity of collective learning (and collective performance) to social stability may thus affect species' vulnerability to human activities that often disrupt social group structures (e.g. [114]). If group performance (e.g. foraging or breeding success) requires a long learning phase, and/or if it involves building inter-dependencies between group members, loss of individuals or changes in the social bonds might have long-lasting effects on the fitness of group members (e.g. extending over several breeding seasons for disrupted pairs of monogamous birds), but it remains to be seen how significant this might be in ecology and conservation compared to other processes and threats [115].

### (c) General conclusions

To sum up, empirical evidence has accumulated for collective learning, i.e. that diverse animal groups are capable of improving their collective performance when repeating a given task. The conditions and mechanisms for collective learning had previously received little focused attention from biologists [5]. We have illustrated here that elements of answers already exist in a vastly scattered, multi-disciplinary literature. We have provided several empirical examples or candidates for collective learning in natural behaviours, yet little is empirically known regarding the actual prevalence of collective learning and even less regarding the relative contributions of the various categories of mechanisms we identified.

Currently, studies explicitly addressing collective learning have mainly been emerging from two converging research avenues: social learning on the one hand (i.e. information transfer between individuals, or ‘learning from’) and collective decision-making on the other. For instance, a lot of attention has been devoted to the links between leadership and individual traits (in particular, individual knowledge), or to the effects of social network structure on information pooling by individual members. By contrast, much less is known about other types of emergent processes, that is, those due to learning *about* others or due to increased complementarity between members, although we cannot exclude that we missed key studies from the extensive and varied literature on group behaviours. We hope our work will help better centralize knowledge about each of these processes in the future.

The importance of the cognitive and/or evolutionary challenges in shaping collective learning capacities are also little understood and may be difficult to address empirically. For these questions, but also more generally for mechanistic studies of the emergence of collective learning, there is vast potential for modelling approaches [23,24,27], including those that incorporate insights developed in artificial learning sciences [72,73]. In particular, more explicit integration of rules for learning *about* others could greatly extend our understanding of the interplay between learning and collective behaviour in animals, as already explored in AI studies [72]. We have highlighted throughout how these concepts expand our understanding of learning beyond current theories on individual and social learning processes, notably including the potentially deep links between learning and social stability. To conclude, there is broad scope for renewed research questions, as much remains to be discovered and understood regarding the importance, distribution, and mechanisms of collective learning across animal groups.

## Data Availability

This article has no additional data.
